# Complete Genome Sequences of *Rice Yellow Mottle Virus* Isolates from the Federal Democratic Republic of Ethiopia

**DOI:** 10.1128/MRA.00589-19

**Published:** 2019-07-25

**Authors:** Mbolarinosy Rakotomalala, Bayuh Belay Abera, Jacqueline Rakotoarisoa, Dawit Alemu, Eugénie Hébrard, Agnès Pinel-Galzi, Denis Fargette

**Affiliations:** a Centre Régional de Recherche Nord-Ouest du FOFIFA, Mahajanga, Madagascar; b Fogera National Rice Research and Training Center, Bahir Dar, Ethiopia; c Direction Générale du FOFIFA, Antananarivo, Madagascar; d Ethiopian Institute for Agricultural Research (EIAR), Ministry of Agriculture, Addis Ababa, Ethiopia; e Interactions Plantes Microorganismes Environnement (IPME), IRD, Cirad, UM, Montpellier, France; KU Leuven

## Abstract

The full-length genomes of two isolates of *Rice yellow mottle virus* from Ethiopia were sequenced. A comparison with 28 sequences from East Africa showed that they clustered within a new strain named S4et, related to the S4mg and S4ug strains found in the Lake Victoria Basin and Madagascar, respectively.

## ANNOUNCEMENT

*Rice yellow mottle virus* (RYMV) is a single-stranded positive-sense RNA virus species of the Sobemovirus genus in the Solemoviridae family (1, 2) that has a ca. 4,450-nucleotide (nt)-long genome organized in five open reading frames (ORFs) (3). RYMV is a major biotic constraint to rice cultivation in Africa and Madagascar (4). In 2012, RYMV was detected in the northwest of the Federal Democratic Republic of Ethiopia, 20 to 25 km east of Lake Tana (5). This is 1,000 km farther north than previously reported in East Africa, at an altitude of 1,800 m, where RYMV is hardly found. The sequences of the 720-nt-long ORF3 gene (encoding the coat protein) from four isolates (Et2, Et3, Et5, and Et21) (5) displayed low genetic divergence (<1.3%). Four additional isolates (Et15, Et19, Et20, and Et105) were collected in paddy fields alongside Lake Tana from rice plants showing symptoms consistent with RYMV infection. Total RNA was extracted from leaves displaying RYMV-like symptoms using the GeneJet plant RNA purification minikit (Thermo Fisher). ORF3 and nearly complete genomes were amplified by reverse transcription-PCR (RT-PCR) with, respectively, one and two pairs of overlapping primers (Table 1), using total RNA as the template (6). After control on 1% agarose gels, purified PCR products were directly sequenced with internal specific primers using an ABI 3730xl DNA analyzer. Two reads per base (in the 3′-to-5′ and 5′-to-3′ directions) led to a sequence accuracy of over 99.9%.

**TABLE 1:** Primers used in RT-PCR to amplify the full genome and ORF3 of *Rice yellow mottle virus*

Primer name	Positions	Primer sequence	Product size (nt)
Full genome			
A_s_	2–22	CAATTGAAGCTAGGAAAGGGAG	2,422
B_AS_	2401–2424	ACTTCGCCGGTTTCGCAGAGGATT	
C_s_	2138–2157	CATGCTGGGAAAAGTGTCTG	2,314
D_AS_	4430–4452	CTCCCCCACCCATCCCGAGAATT	
ORF3			
D_s_	3442–3457	CAAAGATGGCCAGGAA	1,010
D_AS_	4430–4452	CTCCCCCACCCATCCCGAGAATT	

**FIG 1:**
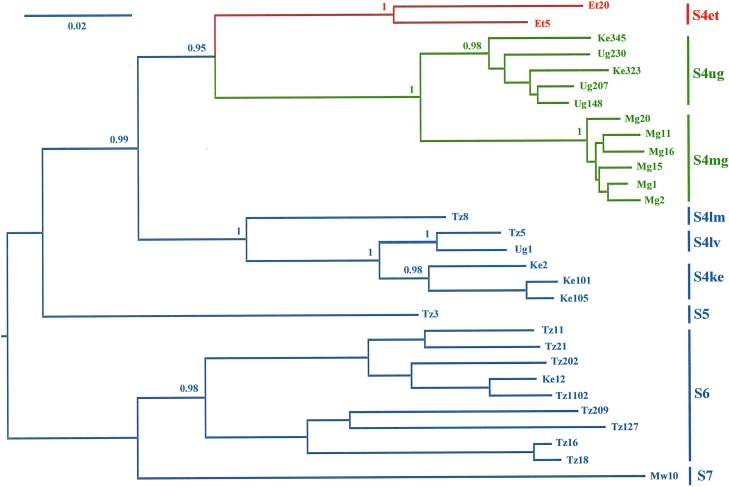
Rooted phylogenetic tree reconstructed by the maximum likelihood method (HKY85 model) from 30 full-length sequences of RYMV from East Africa and Madagascar. The names of the countries are abbreviated as follows: Et (Ethiopia), Ke (Kenya), Mg (Madagascar), Mw (Malawi), Tz (Tanzania), and Ug (Uganda). The strains and accession numbers are as follows: Et5, MH917946; Et20, MH917950; Ke2, MG599276; Ke12, FN432839; Ke101, MG599277; Ke105, MG599278; Ke323, MG599279; Ke345, MG599280; Mw10, MF989228; Mg1, AJ608210; Mg2, AJ608211; Mg11, JX966244; Mg15, JX966245; Mg16, AM883056; Mg20, JX966246; Tz3, AJ608216; Tz5, AJ608217; Tz8, AJ608218; Tz16, JX961551; Tz11, AJ608215; Tz21, JX966247; Tz18, AJ877020; Tz127, AJ876793; Tz202, AM883057; Tz209, AM883058; Tz1102, JX966248; Ug1, KM487710; Ug148, KM487711; Ug207, KM487712; and Ug230, KM487713). Names of the strains are given at the right of the figure. The tips and the branches of the S4et strain are colored in red, while those of the S4ug and S4mg strains are colored in green. The bootstrap support of the strains is shown at the node.

A comparison of the ORF3 of the eight isolates revealed a maximum genetic divergence of 3.4%. The two most divergent isolates (Et5 and Et20), collected 40 km apart in the Dera and Fogera districts within the Tana Basin, were fully sequenced, assembled using SeqMan (Lasergene), and compared to 28 full-length sequences representative of the genetic and geographic diversity of RYMV in East Africa and Madagascar (7–9). The phylogenetic relationships between the 30 sequences were reconstructed through a rooted phylogenetic tree (Fig. 1) with SeaView (10). The recombination events were analyzed with Recombination Detection Program version 4 (RDP4) (11). Only events detected by more than two methods were considered putative recombination events (11).

The full genomes of isolates Et5 and Et20 had ca. 4% nucleotide divergence. Isolates from Ethiopia have an amino acid deletion at position 60 in the coat protein that is shared by isolates of all strains in Africa except those of strains S5 and S6, detected in eastern Tanzania (6), and strain S7, from the south of Malawi (9). Together, the isolates from Ethiopia belong to a new strain referred to as the S4et strain. No recombination event between S4et and any other strain was detected. The closest genetic similarities were with isolates of the S4mg and S4ug strains (ca. 93%). However, the genomes of the S4mg and S4ug strains were more related to each other (ca. 96%) than to that of the S4et strain. Isolates of the S4ug strain have been found in eastern Uganda (7), western Kenya (8), and northeastern Tanzania (12). Isolates of the S4mg strain have occurred only in Madagascar (7). Together, the S4et, S4ug, and S4mg strains comprise a new genetic lineage with high bootstrap support (0.95) (Fig. 1). This lineage has the most eastern and northern geographical extension in Africa.

### Data availability.

The complete sequences of isolates Et5 and Et20 were deposited in GenBank under the accession no. MH917946 and MH917950, respectively. The ORF3 sequences of isolates Et15, Et19, and Et105 were deposited in GenBank under the accession no. MH917947, MH917949, and MH917948, respectively.
